# Association between serum haptoglobin and carotid arterial functions: usefulness of a targeted metabolomics approach

**DOI:** 10.1186/s12933-019-0808-2

**Published:** 2019-01-11

**Authors:** Shiyun Wang, Jie Wang, Rong Zhang, Aihua Zhao, Xiaojiao Zheng, Dandan Yan, Feng Jiang, Wei Jia, Cheng Hu, Weiping Jia

**Affiliations:** 1 Shanghai Diabetes Institute, Shanghai Key Laboratory of Diabetes Mellitus, Shanghai Clinical Center for Diabetes, Shanghai Jiao Tong University Affiliated Sixth People’s Hospital, 600 Yishan Road, Shanghai, 200233 People’s Republic of China; 2 Center for Translational Medicine, Shanghai Key Laboratory of Diabetes Mellitus, Department of Endocrinology and Metabolism, Shanghai Jiao Tong University Affiliated Sixth People’s Hospital, 600 Yishan Road, Shanghai, 200233 People’s Republic of China; 3 Institute for Metabolic Disease, Fengxian Central Hospital Affiliated to Southern Medical University, 6600 Nanfeng Road, Shanghai, 201499 People’s Republic of China

**Keywords:** Metabolites, Haptoglobin, Carotid inter-adventitial diameter, Carotid intima-media thickness, Type 2 diabetes, Non-diabetes mellitus

## Abstract

**Background:**

Serum haptoglobin (Hp) has been closely associated with cardio-cerebrovascular diseases. We investigated a metabolic profile associated with circulating Hp and carotid arterial functions via a targeted metabolomics approach to provide insight into potential mechanisms.

**Methods:**

A total of 240 participants, including 120 patients with type 2 diabetes mellitus (T2DM) and 120 non-diabetes mellitus (non-DM) subjects were recruited in this study. Targeted metabolic profiles of serum metabolites were determined using an AbsoluteIDQ™ p180 Kit (BIOCRATES Life Sciences AG, Innsbruck, Austria). Ultrasound of the bilateral common carotid artery was used to measure intima-media thickness and inter-adventitial diameter. Serum Hp levels were tested by enzyme-linked immunosorbent assay.

**Results:**

Serum Hp levels in T2DM patients and non-DM subjects were 103.40 (72.46, 131.99) mg/dL and 100.20 (53.99, 140.66) mg/dL, respectively. Significant differences of 19 metabolites and 17 metabolites were found among serum Hp tertiles in T2DM patients and non-DM subjects, respectively (*P* < 0.05). Of these, phosphatidylcholine acyl-alkyl C32:2 (PC ae C32:2) was the common metabolite observed in two populations, which was associated with the serum Hp groups and lipid traits (*P* < 0.05). Furthermore, the metabolite ratios of two acidic amino acids, including aspartate to PC ae C32:2 (Asp/PC ae C32:2) and glutamate to PC ae C32:2 (Glu/PC ae C32:2) were correlated with serum Hp, carotid arterial functions and other biochemical index in both populations significantly (*P* < 0.05).

**Conclusions:**

Targeted metabolomics analyses might provide a new insight into the potential mechanisms underlying the association between serum Hp and carotid arterial functions.

**Electronic supplementary material:**

The online version of this article (10.1186/s12933-019-0808-2) contains supplementary material, which is available to authorized users.

## Background

Type 2 diabetes mellitus (T2DM) represents a heterogeneous group of chronic metabolic disorders, and its increasing prevalence has led to an explosion of the global disease burden with increased mortality and morbidity [1, 2]. Systemic metabolic dysfunctions, such as hyperglycaemia, insulin resistance and dyslipidaemia, give rise to an elevated risk of cardio-cerebrovascular diseases, including cerebral infarction, stroke, and heart attacks [3, 4]. The pathogenesis underlying macroangiopathy is complicated and yet not well clarified [5–8]. A group of studies was previously launched to explore the causality underlying the relationship of several novel biomarkers with macroangiopathy risk [9–11], and a causal relationship between serum haptoglobin (Hp) levels and macroangiopathy was observed in Chinese T2DM patients via Mendelian randomization analysis [12].

As a kind of acute-phase response protein, serum Hp is mainly synthesized by hepatocytes and is widely distributed in the circulation in humans [13]. The main physiological function of serum Hp is binding free haemoglobin released from red blood cell lysis and protecting tissues and vessels from oxidative damage [14]. Several studies have reported that increases in circulating Hp levels were observed in T2DM patients and general population with cardio-cerebrovascular diseases [15–17]. Furthermore, it has been proposed that the influence of Hp on the cholesterol level might be related to the ability of Hp to bind apolipoprotein E, which is closely related to lipid metabolism and carotid atherosclerosis [18]. Thus, understanding the mechanisms underlying the interactions involving serum Hp, lipid metabolism and carotid arterial functions might provide a novel clue for the prevention and treatment of cardio-cerebrovascular diseases in T2DM patients and general population.

Recently, metabolomics has been used as a more expeditious and sensitive approach to explore changes in metabolic profiles of chronic metabolic diseases [19, 20]. Metabolites indicate intermediates and end products of metabolic pathways, including carbohydrates, fatty acids, amino acids, pigments, nucleotides, organic acids, vitamins and many other classes of compounds [21]. Changes in metabolites in organisms, tissues or cells are direct indicators of variation in the physiology and pathology of diseases [22]. In current metabolomics research, the three different approaches adopted in the measurement of metabolites are metabolic fingerprinting, metabolite profiling and targeted metabolomics [23].

In the current study, we aimed to investigate whether a targeted metabolomics approach involving a broader spectrum of metabolites may help to identify metabolites associated with the serum Hp levels in Chinese T2DM patients and non-diabetes mellitus (non-DM) subjects. Furthermore, analyses of the altered metabolites and metabolite ratios with the traits of carotid arterial functions, including intima-media thickness (IMT) and inter-adventitial diameter (IAD) of the bilateral common carotid arteries, were performed to provide further insight into the potential mechanisms involved.

## Methods

### Subjects

A total of 240 participants were enrolled in this study, including 120 T2DM patients from the Shanghai Diabetes Institute Inpatient Database and 120 non-DM subjects from the Shanghai Nicheng Cohort Study [24]. T2DM and non-DM were diagnosed by the 1999 WHO criteria following a 75 g oral glucose tolerance test. Written informed consent was obtained from all patients. This current study complied with the Declaration of Helsinki and was approved by the Institutional Review Board of Shanghai Jiao Tong University Affiliated Sixth People’s Hospital, Shanghai, China. Exclusion criteria were subjects with cancer, severe disability, psychiatric disturbances, type 1 diabetes, pregnancy, haemolytic disease, cardiac failure, severe infection, and drug or alcohol addiction. Fasting venous blood samples of all patients were collected and centrifuged, and the separated serum samples were transferred and stored at − 80 °C until assayed.

### Clinical examination

Anthropometric characteristics of all 240 participants, such as age, sex, height, weight, and duration of diabetes (years), were recorded. Body mass index (BMI) was defined as weight (kg)/height^2^ (m^2^). Blood pressure was measured using a mercury sphygmomanometer by an experienced physician, and the mean values of three repeated measurements were recorded. A vascular ultrasound was used to examine the bilateral common carotid arteries. Information on the IMT and IAD was recorded in T2DM patients and IMT was used in non-DM subjects. The mean values of IMT and IAD of the bilateral common carotid arteries were calculated for further analysis. Haemoglobin A1c (HbA1c) levels were measured by a Bio-Rad Variant II haemoglobin testing system (Bio-Rad Laboratories, Hercules, USA). Blood lipid profiles, which included levels of total cholesterol, triglycerides, high-density lipoprotein-cholesterol (HDL-C), and low-density lipoprotein-cholesterol (LDL-C), were tested by a Hitachi 7600-020 Automated Analyzer (Hitachi, Tokyo, Japan).

### Metabolite measurements

The metabolite measurements were conducted via a targeted metabolomics approach by using an AbsoluteIDQ™ p180 Kit (BIOCRATES Life Sciences AG, Innsbruck, Austria) combined with flow injection analysis and liquid chromatography–tandem mass spectrometry. A total of 184 metabolites were detected by this kit, including 40 acylcarnitines (C x:y), 87 glycerophospholipids [14 lyso-phosphatidylcholines (lyso-PCs) (lyso-PC x:y) and 73 phosphatidylcholines (PCs) (35 PC aa x:y and 38 PC ae x:y)], 14 sphingolipids (SM x:y or SM (OH) x:y), 21 amino acids, 21 biogenic amines and 1 hexose. Glycerophospholipids were differentiated according to the presence of ester and ether bonds in the glycerol moiety. Double letters of “aa” or “ae” indicate that two glycerol positions are bound to a fatty acid residue via an ester bond or ether bond, whereas a single letter of “a” or “e” represents only one fatty acid residue bound to the glycerol backbone. The abbreviation “C x:y” is used to indicate the composition of a lipid fatty side chain, where “x” represents the number of carbon atoms and “y” indicates the number of double bonds in the side chain. The assay procedures for measurements and quality control followed the kit manufacturer’s instructions [25]. The samples were analysed using a Waters XEVO™ TQ mass spectrometer (Waters, Manchester, UK) coupled with a Waters ACQUITY(^®^) ultra performance liquid chromatography (UPLC). Biocrates MetIQ™ software (BIOCRATES Life Sciences AG, Innsbruck, Austria) was used to quantify metabolite concentrations and assess the quality of metabolites automatically. Identification and calculations of the metabolite concentration were achieved using internal standards. Metabolite concentrations were reported in the unit of μmol/L (μM), and an Excel file was exported for further statistical analyses. Metabolites below the limit of detection were excluded from further analysis.

### Circulating Hp detection

An enzyme-linked immunosorbent assay (ELISA) was used to measure Hp concentrations by using a Human Haptoglobin Quantikine ELISA kit (R&D Systems, Inc., Minneapolis, USA) following the kit manufacturer’s instructions as described previously [12].

### Statistical analyses

Statistical analyses of the quantitative characteristics were carried out using SAS for Windows (version 9.2; SAS Institute, Cary, NC, USA). Variables were subjected to normality tests, and any skewed quantitative trait data were logarithmically transformed before analysis. Statistical significance of clinical traits and metabolites among three groups of serum Hp levels tertiles (low Hp, middle Hp and high Hp) was determined by one-way analysis of variance (ANOVA) followed by Dunnett’s test to identify group differences at *P* < 0.05. Pearson correlation analysis was performed to evaluate correlations between two variables. Multiple linear regression analysis was used to test the association of metabolites and metabolite ratios with lipid traits after adjusting for confounding factors. All data are shown as n, the mean ± standard deviation or median (interquartile range). A two-tailed *P* value < 0.05 was considered nominally significant. A total of 184 metabolites were measured, and seven were excluded because of low detection quality. Bonferroni correction was applied to adjust for multiple testing (i.e., 177 tests for the analysis of metabolites association). Thus, associations of *P* < adjust *P* (0.05/177) = 2.8 × 10^−4^ for metabolites and serum Hp were considered significant. Comprehensive meta-analysis (version 2.2.057; Biostat, Englewood, New Jersey) was conducted to evaluate combined effects from two populations using a fixed or random effect model after testing for heterogeneity. The Cochran *Q* statistic and the *I*^*2*^ statistic were applied to assess the extent of heterogeneity.

## Results

### Basic clinical features

The median (interquartile range) values of serum Hp levels in T2DM patients and non-DM subjects were 103.40 (72.46, 131.99) mg/dL and 100.20 (53.99, 140.66) mg/dL, respectively. No significant difference was found in serum Hp levels between these two populations (*P* = 0.7315). Anthropometric and clinical characteristics of all the 240 participants grouped by serum Hp level tertiles are described in Table 1.

**Table 1:** Basic clinical characteristics of subjects in this study

Variable	Low Hp	Middle Hp	High Hp	*P* value
T2DM (n = 120)				
Number	39	40	41	–
Age (years)	56.85 ± 11.18	56.88 ± 12.16	58.25 ± 11.46	0.8245
Male/female	30/9	22/18	32/9	*0.0410*
BMI (kg/m^2^)	24.69 ± 4.24	24.63 ± 3.25	24.62 ± 2.89	0.9959
SBP (mmHg)	130 (120, 150)	127 (110, 134)	130 (120, 150)	0.0852
DBP (mmHg)	80 (74, 90)	75 (70, 82)	80 (72, 90)	0.1756
Duration of diabetes (years)	10 (4, 15)	10 (7, 10)	10 (4, 15)	0.8267
HbA1c (%)	8.00 (7.20, 10.20)	8.40 (7.20, 10.30)	8.30 (7.20, 9.70)	0.9903
Total cholesterol (mmol/L)	4.64 (4.28, 5.59)	4.61 (3.78, 5.37)	4.40 (3.87, 4.87)	0.3833
Triglycerides (mmol/L)	1.50 (1.20, 2.20)	1.29 (0.79, 1.93)	1.39 (1.03, 1.65)	0.2756
HDL-C (mmol/L)	1.09 (0.95, 1.20)	0.99 (0.88, 1.24)	1.00 (0.81, 1.10)	0.2181
LDL-C (mmol/L)	2.76 ± 0.85	2.74 ± 0.76	2.65 ± 0.84	0.7488
Carotid IMT (mm)	0.80 (0.70, 0.90)	0.75 (0.65, 0.90)	0.80 (0.70, 0.90)	0.3466
Carotid IAD (mm)	6.17 ± 0.70	6.28 ± 0.78	6.65 ± 0.67	*0.0088*
Serum Hp levels (mg/dL)	54.82 (3.60, 71.58)	103.20 (95.30, 107.27)	151.64 (131.12, 175.55)	< *0.0001*
Non-DM (n = 120)				
Number	39	40	41	–
Age (years)	64.06 ± 4.31	64.10 ± 4.27	64.58 ± 4.15	0.8720
Male/female	17/22	20/20	23/18	0.5378
BMI (kg/m^2^)	24.11 ± 3.27	25.09 ± 3.18	25.64 ± 4.06	0.1522
SBP (mmHg)	125 (120, 139)	129 (122, 138)	129 (123, 138)	0.5074
DBP (mmHg)	81 (78, 87)	81 (78, 83)	82 (80, 87)	0.4709
HbA1c (%)	5.64 ± 0.59	5.61 ± 0.65	5.82 ± 0.51	0.1398
Total cholesterol (mmol/L)	4.36 (4.00, 4.89)	4.49 (3.98, 4.97)	4.40 (4.07, 4.79)	0.8007
Triglycerides (mmol/L)	1.03 (0.80, 1.43)	1.10 (0.89, 1.41)	0.99 (0.80, 1.26)	0.7676
HDL-C (mmol/L)	1.31 (1.09, 1.58)	1.29 (1.17, 1.52)	1.22 (1.14, 1.41)	0.6175
LDL-C (mmol/L)	2.53 (2.25, 2.89)	2.56 (2.15, 2.97)	2.61 (2.28, 2.91)	0.7962
Carotid IMT (mm)	0.64 ± 0.10	0.65 ± 0.09	0.66 ± 0.08	0.4667
Serum Hp levels (mg/dL)	39.48 (17.23, 51.45)	99.41 (92.96, 113.38)	167.67 (140.35, 209.73)	< *0.0001*

Data are shown as n or the mean ± standard deviation or median (interquartile range)

*T2DM* type 2 diabetes mellitus, *Non-DM* non-diabetes mellitus, *Hp* haptoglobin, *BMI* body mass index, *SBP* systolic blood pressure, *DBP* diastolic blood pressure, *HDL-C* high-density lipoprotein-cholesterol, *LDL-C* low-density lipoprotein-cholesterol, *IMT* intima-media thickness, *IAD* inter-adventitial diameter

*P* values < 0.05 are shown in italic

**Fig. 1:**
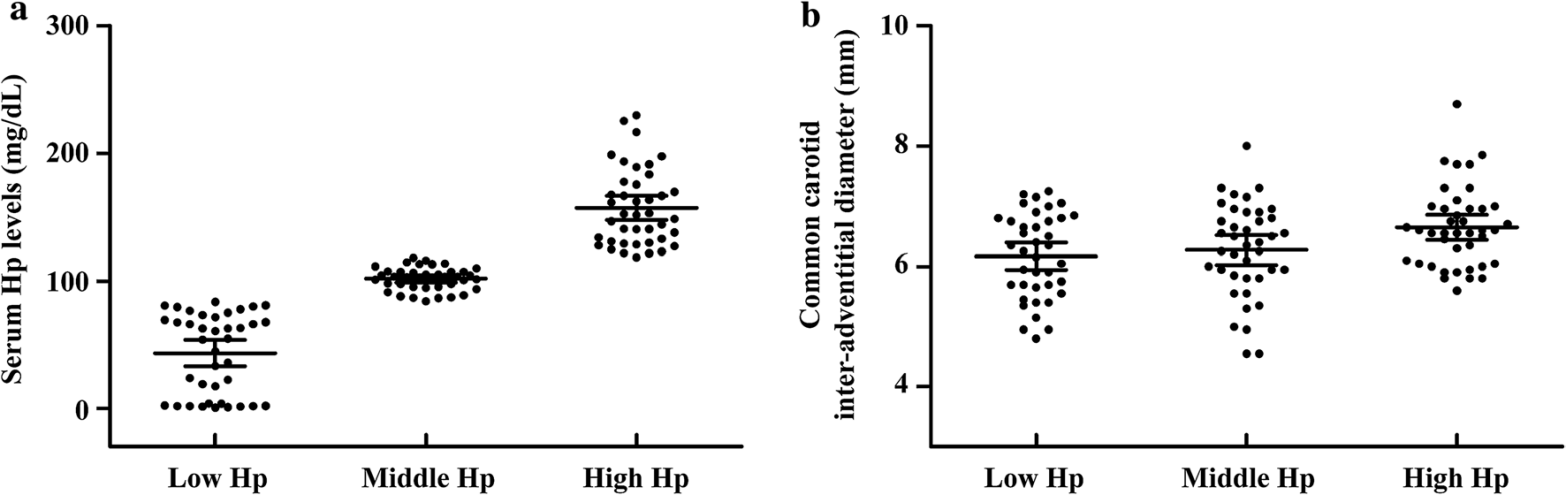
Serum haptoglobin levels and common carotid inter-adventitial diameter in T2DM patients. *T2DM* type 2 diabetes mellitus, *Hp* haptoglobin. Dot plot **a** shows a comparison of serum Hp levels among Hp tertiles, *P* < 0.0001. Dot plot **b** shows a comparison of common carotid artery inter-adventitial diameter levels among Hp tertiles, *P *= 0.0090. *P* values were determined by one-way analysis of variance. The Hp and carotid artery inter-adventitial diameter levels are shown in dot plots; the mean is indicated by the middle black solid line. The 95% confidence intervals are shown by the bottom and top black solid lines, respectively

In T2DM patients, the median (interquartile range) values of serum Hp concentrations in low Hp, middle Hp and high Hp groups were 54.82 (3.60, 71.58) mg/dL, 103.20 (95.30, 107.27) mg/dL and 151.64 (131.12, 175.55) mg/dL, respectively. After comparison among groups, significant differences were found in sex (*P* = 0.0410), carotid IAD (*P* = 0.0088) and serum Hp levels (*P* < 0.0001) (Fig. 1). Furthermore, after adjusting for age, sex, BMI, blood pressure, duration of diabetes and HbA1c levels, the carotid IAD parameter was shown to have a significant relationship with serum Hp tertiles (*P* = 0.0042) (see Additional file 1: Table S1).

In non-DM subjects, the median (interquartile range) values of serum Hp concentrations in low Hp, middle Hp and high Hp groups were 39.48 (17.23, 51.45) mg/dL, 99.41 (92.96, 113.38) mg/dL and 167.67 (140.35, 209.73) mg/dL, respectively, with a significant difference of serum Hp levels among three groups (*P* < 0.0001) (see Additional file 1: Figure S1). After adjusting for age, sex, BMI, blood pressure and HbA1c levels, no parameters was shown to have a significant relationship with serum Hp tertiles (*P* > 0.05) (see Additional file 1: Table S2).

### Association between blood metabolites and serum Hp levels

A total of 184 metabolites were measured, and seven were excluded because of low detection quality. As a result, significant differences of 19 metabolites (2 acylcarnitines, 1 lyso-PCs, 12 PCs, and 4 amino acids) and 17 metabolites (5 acylcarnitines, 2 lyso-PCs, 7 PCs, and 3 amino acids) were found among the Hp level groups from the remaining 177 metabolites in T2DM patients and non-DM subjects, respectively (*P* < 0.05) (Table 2). After further linear regression analysis, 41 metabolites (1 acylcarnitine, 3 lyso-PCs, 33 PCs and 4 amino acids) and 17 metabolites (3 acylcarnitine, 1 lyso-PCs, 10 PCs and 3 amino acids) were found to be associated with serum Hp levels (*P* < 0.05) in T2DM patients and non-DM subjects, respectively (see Additional file 1: Table S3).

**Table 2:** Blood metabolites in subjects grouped by serum Hp levels

Metabolites	Low Hp	Middle Hp	High Hp	*P* value
T2DM (n = 120)				
Acylcarnitines (μM)				
C12:1	0.142 ± 0.042	0.148 ± 0.044	0.116 ± 0.032	0.0017
C14:1	0.241 ± 0.064	0.229 ± 0.067	0.201 ± 0.048	0.0119
Lyso-phosphatidylcholines (μM)				
Lyso-PC a C20:3	1.284 ± 0.423	1.050 ± 0.319	1.064 ± 0.332	0.0158
Phosphatidylcholines (μM)				
PC aa C32:1	5.437 ± 2.670	4.678 ± 1.994	4.128 ± 1.775	0.0398
PC aa C34:1	109.683 ± 37.529	99.877 ± 34.435	89.110 ± 23.902	0.0117
PC aa C34:2	323.235 ± 80.457	308.206 ± 90.611	283.969 ± 72.902	0.0456
PC aa C36:1	22.898 ± 6.344	21.283 ± 4.553	19.615 ± 6.216	0.0041
PC aa C40:4	2.093 ± 0.646	1.881 ± 0.541	1.809 ± 0.594	0.0458
PC ae C32:1	1.329 ± 0.294	1.281 ± 0.268	1.171 ± 0.245	0.0364
PC ae C32:2	0.335 ± 0.088	0.301 ± 0.062	0.281 ± 0.058	0.0094
PC ae C34:1	3.305 ± 0.712	3.254 ± 0.733	2.902 ± 0.559	0.0270
PC ae C36:0	0.577 ± 0.180	0.546 ± 0.128	0.470 ± 0.098	0.0055
PC ae C36:1	4.953 ± 0.948	4.642 ± 0.925	4.417 ± 0.814	0.0327
PC ae C40:5	2.140 ± 0.418	2.034 ± 0.527	1.871 ± 0.374	0.0329
PC ae C42:2	0.333 ± 0.080	0.307 ± 0.082	0.287 ± 0.060	0.0443
Amino acids (μM)				
His	115.000 ± 15.508	111.528 ± 15.191	105.444 ± 14.510	0.0183
Lys	330.411 ± 57.377	306.053 ± 61.629	300.033 ± 54.178	0.0255
Trp	65.594 ± 14.239	59.247 ± 13.020	58.258 ± 10.826	0.0238
Tyr	89.152 ± 19.114	84.488 ± 17.566	79.436 ± 16.558	0.0494
Non-DM (n = 120)				
Acylcarnitines (μM)				
C16:1-OH	0.018 ± 0.004	0.010 ± 0.002	0.013 ± 0.002	0.0440
C2	6.327 ± 2.720	5.684 ± 1.558	6.773 ± 2.023	0.0385
C3	0.334 ± 0.109	0.299 ± 0.124	0.386 ± 0.191	0.0294
C4	0.211 ± 0.075	0.184 ± 0.082	0.225 ± 0.090	0.0209
C6/C4:1-DC	0.085 ± 0.038	0.083 ± 0.058	0.113 ± 0.074	0.0240
Lyso-phosphatidylcholines (μM)				
LysoPC a C17:0	1.302 ± 0.361	1.208 ± 0.318	1.115 ± 0.326	0.0463
LysoPC a C24:0	0.371 ± 0.121	0.312 ± 0.112	0.307 ± 0.120	0.0450
Phosphatidylcholines (μM)				
PC aa C32:3	0.260 ± 0.047	0.239 ± 0.061	0.225 ± 0.059	0.0087
PC aa C42:2	0.276 ± 0.062	0.289 ± 0.116	0.243 ± 0.070	0.0326
PC ae C32:2	0.475 ± 0.098	0.453 ± 0.131	0.424 ± 0.089	0.0602
PC ae C36:2	7.123 ± 1.446	6.882 ± 2.063	6.309 ± 1.124	0.0304
PC ae C38:0	1.141 ± 0.298	1.062 ± 0.377	0.969 ± 0.252	0.0471
PC ae C40:1	1.286 ± 0.246	1.164 ± 0.263	1.101 ± 0.189	0.0071
PC ae C42:1	0.372 ± 0.093	0.347 ± 0.091	0.321 ± 0.071	0.0197
Amino acids (μM)				
Asp	32.646 ± 5.990	31.854 ± 4.664	36.137 ± 6.343	0.0034
Trp	75.043 ± 8.173	71.877 ± 9.742	68.875 ± 11.423	0.0244
Ac-Orn	2.189 ± 0.159	2.239 ± 0.001	1.596 ± 0.941	0.0185

Data are shown as the mean ± standard deviation. *P* values < 0.05 are shown in table

*T2DM* type 2 diabetes mellitus, * Non-DM * non-diabetes mellitus, *Hp* haptoglobin, *His* histidine, *Lys* lysine, *Trp* tryptophan, *Tyr* tyrosine, *Asp* aspartate, *Ac-Orn* acyl-ornithine

### Selected metabolites associated with blood lipid traits

**Fig. 2:**
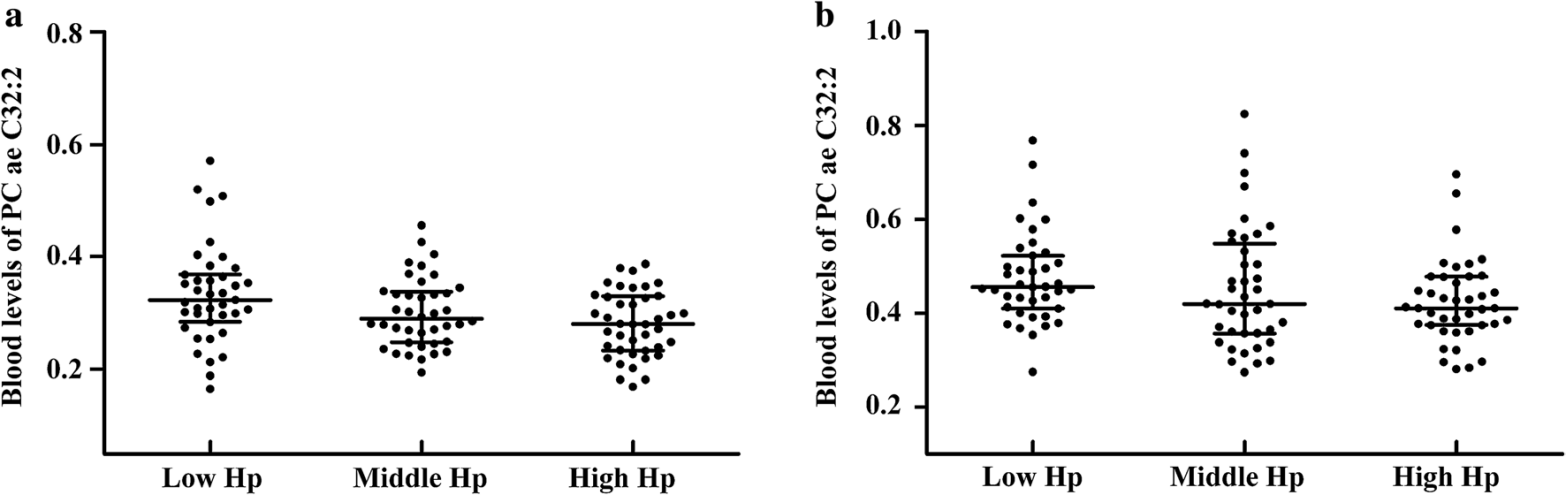
Blood levels of metabolite PC ae C32:2 in subjects grouped by serum Hp levels. *Hp* haptoglobin, *T2DM* type 2 diabetes mellitus,* Non-DM* non-diabetes mellitus. Dot plot **a** shows the association of blood metabolite PC ae C32:2 with serum Hp tertiles in T2DM patients, *P* = 0.0005, β ± SE = − 0.028 ± 0.008. Dot plot **b** shows the association of blood metabolite PC ae C32:2 with serum Hp tertiles in non-DM patients, *P* = 0.0390, β ± SE = − 0.026 ± 0.012. *P* values and beta values were determined by multiple linear regression adjusting for age, sex, body mass index, total cholesterol, triglycerides, high-density lipoprotein-cholesterol, low-density lipoprotein-cholesterol, inter-adventitial diameter and/or carotid intima-media thickness. The PC ae C32:2 levels are shown in dot plots; the median is indicated by the middle black solid line. The lower and upper quartiles are shown by the bottom and top black solid lines, respectively

Multiple linear regression analysis was conducted to investigate the association of the altered metabolites with traits of blood lipids and carotid arterial functions (carotid IMT and carotid IAD). After adjusting for age, sex and BMI, 10 metabolites (1 lyso-PC, 7 PCs and 2 amino acids) and 10 metabolites (4 lyso-PC, 5 PCs and 1 amino acids) were found to be associated with the serum Hp groups and lipid traits in T2DM patients (Table 3) and non-DM subjects (Table 4), respectively. Among them, phosphatidylcholine acyl-alkyl C32:2 (PC ae C32:2) was the common metabolite associated with the serum Hp groups and lipid traits in both two populations. In T2DM patients, PC ae C32:2 was significantly correlated with total cholesterol (*P* = 0.0390), the serum Hp groups (*P* = 0.0005) and carotid IAD (*P* = 0.0182). In non-DM subjects, PC ae C32:2 was associated with HDL-C levels (*P* = 0.0133) and the serum Hp groups (*P* = 0.0390) significantly. The combined analysis of two populations by a meta-analysis revealed that PC ae C32:2 was correlated with the serum Hp groups (β ± SE = − 0.028 ± 0.007, *P* = 3.01 × 10^−5^). The Cochran *Q* test showed no heterogeneity in the relationships between PC ae C32:2 and serum Hp from T2DM patients and non-DM subjects (*I*^*2*^ = 0, *P* = 0.874). The levels of PC ae C32:2 in T2DM patients and non-DM subjects grouped by serum Hp levels are shown in Fig. 2.

**Table 3:** Blood metabolites correlated with blood lipids and serum Hp levels in T2DM patients

Metabolites	Total cholesterol	Triglycerides	HDL-C	LDL-C	Serum Hp	Carotid IMT	Carotid IAD
Acylcarnitines (μM)							
C16-OH	0.017 ± 0.011	− 0.002 ± 0.002	− 0.011 ± 0.005	− 0.008 ± 0.006	− 0.001 ± 0.001	0.012 ± 0.004	− 0.002 ± 0.007
	0.1099	0.4393	*0.0270*	0.1690	*0.0452*	*0.0074*	0.7911
Lyso-phosphatidylcholines (μM)							
Lyso-PC a C20:3	− 0.316 ± 1.027	0.834 ± 0.233	1.073 ± 0.485	− 0.202 ± 0.580	− 0.092 ± 0.041	− 0.173 ± 0.404	0.511 ± 0.693
	0.7588	*0.0005*	*0.0290*	0.7286	*0.0272*	0.6698	0.4621
Phosphatidylcholines (μM)							
PC aa C32:0	10.436 ± 4.062	1.295 ± 0.921	1.681 ± 1.918	− 2.026 ± 2.294	− 0.356 ± 0.163	2.011 ± 1.597	3.939 ± 2.741
	*0.0116*	0.1626	0.3828	0.3791	*0.0308*	0.2106	0.1536
PC aa C32:1	− 4.69 ± 5.368	5.805 ± 1.217	6.355 ± 2.534	5.062 ± 3.032	− 0.458 ± 0.215	− 0.323 ± 2.11	2.788 ± 3.622
	0.3843	< *0.0001*	*0.0137*	0.0979	*0.0356*	0.8787	0.4432
PC aa C34:1	− 11.594 ± 75.657	92.794 ± 17.157	89.522 ± 35.716	57.696 ± 42.730	− 7.002 ± 3.033	− 2.744 ± 29.739	34.784 ± 51.050
	0.8785	< *0.0001*	*0.0137*	0.1798	*0.0229*	0.9267	0.4971
PC aa C34:4	0.095 ± 0.634	0.617 ± 0.144	0.824 ± 0.299	0.145 ± 0.358	− 0.054 ± 0.025	− 0.193 ± 0.249	− 0.092 ± 0.428
	0.8806	< *0.0001*	*0.0070*	0.6861	*0.0370*	0.4397	0.8303
PC aa C36:3	14.350 ± 36.596	49.046 ± 8.299	69.044 ± 17.276	− 5.324 ± 20.669	− 3.283 ± 1.467	− 9.846 ± 14.385	10.639 ± 24.693
	0.6957	< *0.0001*	*0.0001*	0.7972	*0.0273*	0.4951	0.6674
PC ae C32:1	0.972 ± 0.740	− 0.094 ± 0.168	0.437 ± 0.349	0.072 ± 0.418	− 0.078 ± 0.030	− 0.054 ± 0.291	0.964 ± 0.499
	0.1918	0.5766	0.2136	0.8629	*0.0095*	0.8543	0.0561
PC ae C32:2	0.411 ± 0.196	− 0.033 ± 0.045	0.081 ± 0.093	− 0.136 ± 0.111	− 0.028 ± 0.008	− 0.073 ± 0.077	0.318 ± 0.133
	*0.0390*	0.4613	0.3837	0.2241	*0.0005*	0.3475	*0.0182*
PC ae C34:1	2.502 ± 1.734	0.505 ± 0.393	1.905 ± 0.819	− 0.554 ± 0.979	− 0.171 ± 0.070	0.759 ± 0.682	1.971 ± 1.170
	0.1519	0.2021	*0.0218*	0.5728	*0.0155*	0.2678	0.0950
PC ae C36:5	10.917 ± 4.572	− 1.698 ± 1.037	2.473 ± 2.158	− 2.333 ± 2.582	− 0.389 ± 0.183	− 2.415 ± 1.797	0.752 ± 3.085
	*0.0187*	0.1044	0.2544	0.3683	*0.0360*	0.1818	0.8079
PC ae C38:6	5.671 ± 3.030	− 0.272 ± 0.687	2.976 ± 1.431	− 0.609 ± 1.711	− 0.252 ± 0.121	− 1.226 ± 1.191	0.707 ± 2.045
	0.0640	0.6927	*0.0399*	0.7227	*0.0407*	0.3058	0.7303
PC ae C40:5	2.559 ± 1.247	− 0.166 ± 0.283	0.694 ± 0.589	− 0.934 ± 0.704	− 0.111 ± 0.050	− 0.314 ± 0.490	0.182 ± 0.841
	*0.0426*	0.5596	0.2413	0.1877	*0.0286*	0.5231	0.8287
Amino acids (μM)							
His	− 150.758 ± 41.991	37.965 ± 9.523	39.971 ± 19.823	65.304 ± 23.716	− 4.080 ± 1.683	6.756 ± 16.506	− 17.531 ± 28.334
	*0.0005*	*0.0001*	*0.0463*	*0.0069*	*0.0170*	0.6831	0.5374
Phe	− 36.314 ± 54.241	13.975 ± 12.301	− 7.065 ± 25.606	33.892 ± 30.634	− 4.385 ± 2.175	57.330 ± 21.321	41.455 ± 36.600
	0.5046	0.2584	0.7831	0.2711	*0.0462*	*0.0083*	0.2599

Multiple linear regression analysis was used to perform the analysis after adjusting for age, sex and body mass index with total cholesterol, triglycerides, HDL-C, LDL-C, serum Hp level tertiles, carotid IMT and IAD as independent variables. β ± SE and *P* values are shown in table

*T2DM* type 2 diabetes mellitus, *Hp* haptoglobin, *His* histidine, *Phe* phenylalanine, *HDL-C* high-density lipoprotein-cholesterol, *LDL-C* low-density lipoprotein-cholesterol, *IMT* intima-media thickness, *IAD* inter-adventitial diameter

*P* values < 0.05 are shown in italic

**Table 4:** Blood metabolites correlated with blood lipids and serum Hp levels in non-DM subjects

Metabolites	Total cholesterol	Triglycerides	HDL-C	LDL-C	Serum Hp	Carotid IMT
Lyso-phosphatidylcholines (μM)						
LysoPC a C16:0	− 494.124 ± 89.651	39.362 ± 16.748	133.887 ± 36.720	310.854 ± 52.472	− 5.264 ± 2.417	25.137 ± 34.301
	< *0.0001*	*0.0207*	*0.0004*	<*0.0001*	*0.0317*	0.4653
LysoPC a C17:0	− 3.953 ± 1.356	0.401 ± 0.253	1.140 ± 0.555	2.706 ± 0.793	− 0.088 ± 0.037	− 0.303 ± 0.519
	*0.0043*	0.1160	*0.0425*	*0.0009*	*0.0183*	0.5606
LysoPC a C18:2	52.100 ± 26.226	− 2.782 ± 4.899	− 0.547 ± 10.742	− 28.897 ± 15.350	− 1.675 ± 0.707	− 5.400 ± 10.034
	*0.0496*	0.5714	0.9595	0.0626	*0.0197*	0.5916
LysoPC a C24:0	− 1.237 ± 0.515	0.239 ± 0.096	0.724 ± 0.211	0.648 ± 0.301	− 0.031 ± 0.014	0.191 ± 0.197
	*0.0181*	*0.0145*	*0.0009*	*0.0339*	*0.0302*	0.3344
Phosphatidylcholines (μM)						
PC ae C32:2	0.007 ± 0.463	0.029 ± 0.087	0.478 ± 0.190	0.114 ± 0.271	− 0.026 ± 0.012	− 0.229 ± 0.177
	0.9886	0.7380	*0.0133*	0.6752	*0.0390*	0.2000
PC ae C34:0	− 0.158 ± 0.737	0.284 ± 0.138	0.666 ± 0.302	0.075 ± 0.431	− 0.041 ± 0.020	− 0.080 ± 0.282
	0.8312	*0.0418*	*0.0295*	0.8621	*0.0441*	0.7761
PC ae C34:3	15.585 ± 3.858	− 1.788 ± 0.721	1.492 ± 1.580	− 6.850 ± 2.258	− 0.230 ± 0.104	− 2.803 ± 1.476
	*0.0001*	*0.0148*	0.3473	*0.0031*	*0.0292*	0.0605
PC ae C36:2	29.048 ± 6.159	− 0.149 ± 1.151	− 2.674 ± 2.523	− 15.795 ± 3.605	− 0.338 ± 0.166	− 2.884 ± 2.356
	< *0.0001*	0.8969	0.2915	< *0.0001*	*0.0446*	0.2238
PC ae C40:1	2.153 ± 0.872	0.357 ± 0.163	0.862 ± 0.357	− 0.748 ± 0.510	− 0.074 ± 0.024	− 0.253 ± 0.334
	*0.0152*	*0.0306*	*0.0176*	0.1456	*0.0020*	0.4491
Amino acids (μM)						
Gln	865.339 ± 430.321	− 124.218 ± 80.390	− 303.854 ± 176.254	− 428.871 ± 251.864	− 32.233 ± 11.602	− 127.624 ± 164.646
	*0.0469*	0.1254	0.0877	0.0916	*0.0065*	0.4400

Multiple linear regression analysis was used to perform the analysis after adjusting for age, sex and body mass index with total cholesterol, triglycerides, HDL-C, LDL-C, serum Hp level tertiles and carotid IMT as independent variables. β ± SE and *P* values are shown in table

*Non-DM* non-diabetes mellitus, *Hp* haptoglobin, *Gln* Glutamine, *HDL-C* high-density lipoprotein-cholesterol, *LDL-C* low-density lipoprotein-cholesterol, *IMT* intima-media thickness

*P* values < 0.05 are shown in italic

### Metabolite ratios associated with clinical traits

In addition, changes in metabolite ratios might reflect biological situations such as changes in enzyme activities or imbalances in metabolic pathways [26]. The metabolite ratio of valine to PC ae C32:2 (Val/PC ae C32:2) was previously reported to be associated with an increased risk of type 2 diabetes and measures of insulin secretion and resistance [27]. In the current study, the ratios of 21 amino acids to PC ae C32:2 were compared to evaluate the association between serum Hp levels and clinical traits.

We found the metabolite ratios of 5 aliphatic amino acids (asparagine, aspartate, glutamate, isoleucine and valine) to PC ae C32:2 were associated with blood lipids, the serum Hp groups, and carotid IMT and/or IAD after adjusting for age, sex and BMI in T2DM patients (*P* < 0.05) (Table 5 and Additional file 1: Table S4). In non-DM subjects, the metabolite ratios of three amino acids (aspartate, glutamate, and phenylalanine) to PC ae C32:2 were associated with blood lipids, the serum Hp groups and carotid IMT after adjusting for age, sex and BMI (*P* < 0.05) (Table 6 and Additional file 1: Table S5).

**Table 5:** Metabolite ratios correlated with blood lipids and serum Hp levels in T2DM patients

Metabolite ratios	Total cholesterol	Triglycerides	HDL-C	LDL-C	Serum Hp	Carotid IMT	Carotid IAD
Asn/PC ae C32:2	− 0.949 ± 0.290	0.061 ± 0.066	− 0.006 ± 0.137	0.277 ± 0.164	0.033 ± 0.012	0.344 ± 0.114	− 0.230 ± 0.196
	*0.0015*	0.3555	0.9626	0.0942	*0.0059*	*0.0032*	0.2433
Asp/PC ae C32:2	− 0.723 ± 0.367	0.047 ± 0.083	− 0.153 ± 0.173	0.290 ± 0.207	0.041 ± 0.015	0.361 ± 0.144	− 0.516 ± 0.248
	*0.0513*	0.5726	0.3789	0.1651	*0.0059*	*0.0138*	*0.0396*
Glu/PC ae C32:2	− 1.121 ± 0.400	0.218 ± 0.091	− 0.113 ± 0.189	0.554 ± 0.226	0.039 ± 0.016	0.252 ± 0.157	− 0.817 ± 0.270
	*0.0061*	*0.0183*	0.5520	*0.0160*	*0.0160*	0.1125	*0.0031*
Ile/PC ae C32:2	− 1.084 ± 0.329	0.241 ± 0.075	− 0.037 ± 0.156	0.473 ± 0.186	0.031 ± 0.013	0.170 ± 0.129	− 0.534 ± 0.222
	*0.0014*	*0.0017*	0.8101	*0.0124*	*0.0222*	0.1933	*0.0180*
Val/PC ae C32:2	− 1.011 ± 0.300	0.189 ± 0.068	− 0.071 ± 0.142	0.499 ± 0.169	0.031 ± 0.012	0.183 ± 0.118	− 0.569 ± 0.202
	*0.0010*	*0.0064*	0.6197	*0.0039*	*0.0116*	0.1241	*0.0059*

Multiple linear regression analysis was used to perform the analysis after adjusting for age, sex and body mass index with total cholesterol, triglycerides, HDL-C, LDL-C, serum Hp level tertiles, carotid IMT and IAD as independent variables. β ± SE and *P* values are shown in table

*T2DM* type 2 diabetes mellitus, *Asn* asparagine, *Asp* aspartate, *Glu* glutamic acid, *Ile* isoleucine, *Val* valine, *Hp* haptoglobin, *HDL-C* high-density lipoprotein-cholesterol, *LDL-C* low-density lipoprotein-cholesterol, *IMT* intima-media thickness, *IAD* inter-adventitial diameter

*P* values < 0.05 are shown in italic

**Table 6:** Metabolite ratios correlated with blood lipids and serum Hp levels in non-DM subjects

Metabolite ratios	Total cholesterol	Triglycerides	HDL-C	LDL-C	Serum Hp	Carotid IMT
Asp/PC ae C32:2	0.028 ± 0.538	− 0.072 ± 0.101	− 0.605 ± 0.220	− 0.254 ± 0.315	0.048 ± 0.015	0.362 ± 0.206
	0.9592	0.4748	*0.0071*	0.4212	*0.0012*	*0.0820*
Glu/PC ae C32:2	− 0.830 ± 0.584	0.020 ± 0.109	− 0.442 ± 0.239	0.317 ± 0.342	0.043 ± 0.016	0.386 ± 0.223
	0.1585	0.8514	*0.0673*	0.3559	*0.0071*	*0.0870*
Phe/PC ae C32:2	− 0.311 ± 0.407	− 0.052 ± 0.076	− 0.493 ± 0.167	0.062 ± 0.238	0.029 ± 0.011	0.351 ± 0.156
	0.4470	0.4945	*0.0039*	0.7948	*0.0094*	*0.0267*

Multiple linear regression analysis was used to perform the analysis after adjusting for age, sex and body mass index with total cholesterol, triglycerides, HDL-C, LDL-C, serum Hp level tertiles and carotid IMT as independent variables. β ± SE and *P* values are shown in table

*Non-DM* non-diabetes mellitus, *Asp* aspartate, *Glu* glutamic acid, *Phe* phenylalanine, *Hp* haptoglobin, *HDL-C* high-density lipoprotein-cholesterol, *LDL-C* low-density lipoprotein-cholesterol, *IMT* intima-media thickness

*P* values < 0.05 or approximately 0.05 are shown in italic

**Fig. 3:**
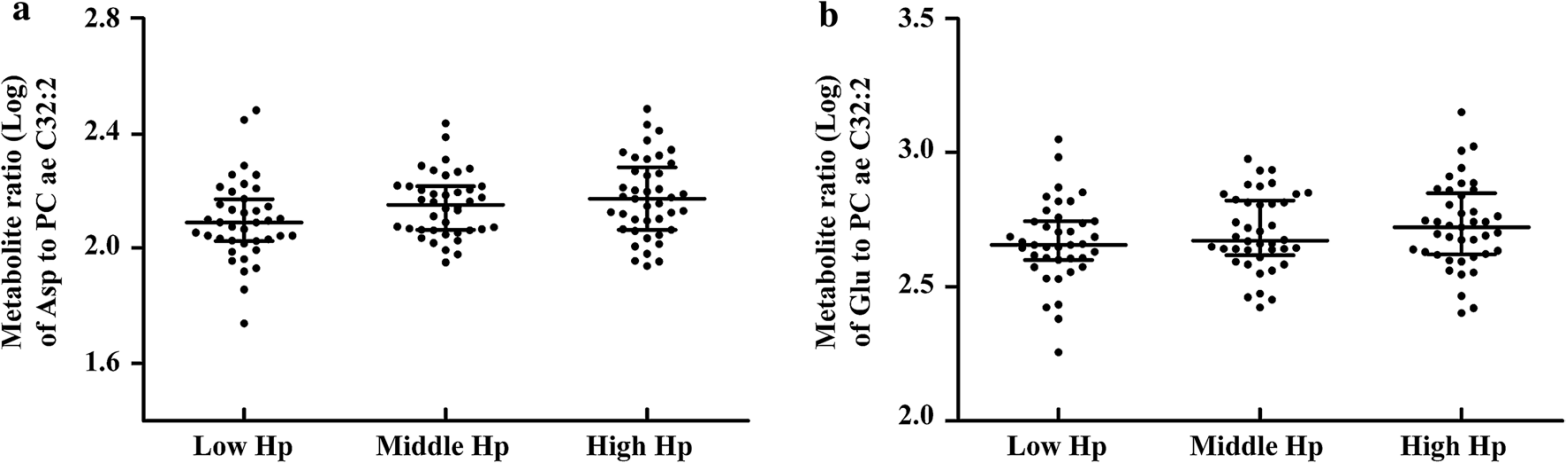
Metabolite ratios of two acidic amino acids to PC ae C32:2 in T2DM patients. *T2DM* type 2 diabetes mellitus, *Hp* haptoglobin, *Asp* aspartate, *Glu* glutamate. Dot plot **a** shows the association of blood metabolite ratio Asp to PC ae C32:2 (Log transformed) with serum Hp tertiles, *P* = 0.0059, β ± SE = 0.041 ± 0.015. Dot plot **b** shows the association of blood metabolite ratio Glu to PC ae C32:2 (Log transformed) with serum Hp tertiles, *P* = 0.0160, β ± SE = 0.039 ± 0.016. *P* values and beta values were determined by multiple linear regression adjusting for age, sex, body mass index, total cholesterol, triglycerides, high-density lipoprotein-cholesterol, low-density lipoprotein-cholesterol, carotid intima-media thickness and inter-adventitial diameter. Metabolite ratios (Log transformed) are shown in dot plots; the median is indicated by the middle black solid line. The lower and upper quartiles are shown by the bottom and top black solid lines, respectively

**Fig. 4:**
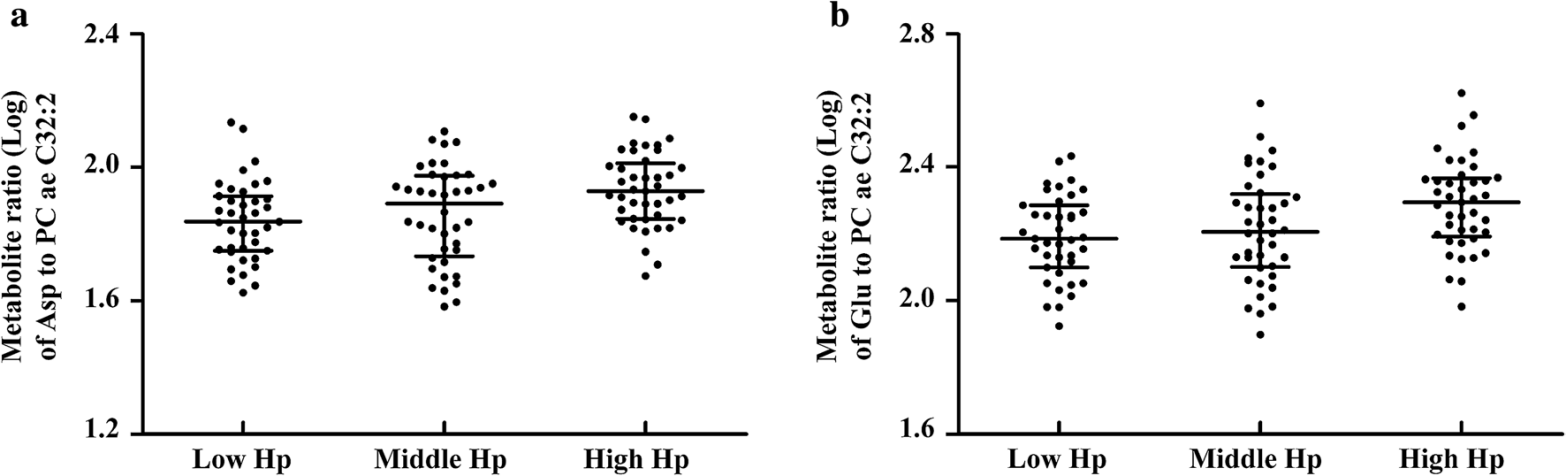
Metabolite ratios of two acidic amino acids to PC ae C32:2 in non-DM subjects. *Non-DM* non-diabetes mellitus, *Hp* haptoglobin, *Asp* aspartate, *Glu* glutamate. Dot plot **a** shows the association of blood metabolite ratio Asp to PC ae C32:2 (Log transformed) with serum Hp tertiles, *P* = 0.0012, β ± SE = 0.048 ± 0.015. Dot plot **b** shows the association of blood metabolite ratio Glu to PC ae C32:2 (Log transformed) with serum Hp tertiles, *P* = 0.0071, β ± SE = 0.043 ± 0.016. *P* values and beta values were determined by multiple linear regression adjusting for age, sex, body mass index, total cholesterol, triglycerides, high-density lipoprotein-cholesterol, low-density lipoprotein-cholesterol and carotid intima-media thickness. Metabolite ratios (Log transformed) are shown in dot plots; the median is indicated by the middle black solid line. The lower and upper quartiles are shown by the bottom and top black solid lines, respectively

Of these, the metabolite ratios of two acidic amino acids, including aspartate to PC ae C32:2 (Asp/PC ae C32:2) and glutamate to PC ae C32:2 (Glu/PC ae C32:2) were the common significant ratios observed in both populations. The combined analysis of two populations by a meta-analysis revealed that Asp/PC ae C32:2 and Glu/PC ae C32:2 were correlated with the serum Hp groups (β ± SE = 0.045 ± 0.010, *P* = 1.45 × 10^−5^; β ± SE = 0.041 ± 0.011, *P* = 2.38 × 10^−4^), respectively. The Cochran *Q* test showed no heterogeneity in the relationships of Asp/PC ae C32:2 and Glu/PC ae C32:2 with serum Hp from T2DM patients and non-DM subjects (*I*^*2*^ = 0, *P* = 0.740; *I*^*2*^ = 0, *P* = 0.860), respectively. The ratios of Asp/PC ae C32:2 and Glu/PC ae C32:2 in T2DM patients and non-DM subjects grouped by serum Hp levels are shown in Figs. 3, 4, respectively.

## Discussion

In the current study, serum Hp levels in T2DM patients and non-DM subjects were 103.40 (72.46, 131.99) mg/dL and 100.20 (53.99, 140.66) mg/dL, respectively. Significant differences of 19 metabolites and 17 metabolites were found among serum Hp tertiles in T2DM patients and non-DM subjects, respectively. Among them, the phosphatidylcholine PC ae C32:2 was shown to be notably correlated with the serum Hp groups and lipid traits in both two populations. Furthermore, the metabolite ratios of two acidic amino acids, including aspartate to PC ae C32:2 (Asp/PC ae C32:2) and glutamate to PC ae C32:2 (Glu/PC ae C32:2) were associated with serum Hp, carotid arterial functions and other biochemical index significantly. These findings might provide a new insight into the potential mechanisms underlying the association between serum Hp and carotid arterial functions.

An acute-phase glycoprotein, Hp was identified in 1938 for the first time. This protein is produced mostly by hepatocytes and is widely distributed in the circulation in humans. The main physiological function of Hp is binding free haemoglobin with high affinity to form a stable haemoglobin–haptoglobin (Hb–Hp) complex, thereby protecting tissues and vessels from oxidative damage. In the range of 50–300 mg/dL, the circulating level of Hp ranks only behind the predominant plasma proteins albumin and immunoglobulins [13]. Several studies reported that circulating Hp levels were correlated with the progression of cardio-cerebrovascular diseases in T2DM patients and general population [16, 17]. Furthermore, a causal relationship between serum Hp levels and macroangiopathy was previously observed in Chinese T2DM patients via Mendelian randomization analysis [12]. Therefore, it is important to study the mechanisms underlying the interactions involving serum Hp and carotid arterial functions to provide a novel insight into the prevention and treatment for cardio-cerebrovascular diseases.

The advent of metabonomics, a new rapidly developing technology, offers a more powerful and sensitive means to explain the aetiology of complex chronic diseases [19, 22]. Glycerophospholipids including PCs and lyso-PCs have recently gained attention as potential biomarkers of cardiovascular disease [28, 29]. PCs not only play a key role as a component of cellular membranes but also act as a reservoir for fatty acids [30]. Lyso-PCs mostly come from the partial hydrolysis of PCs catalysed mainly by phospholipase A1 (PLA1) and phospholipase A2 (PLA2), lecithin–cholesterol acyltransferase (LCAT) activity or hepatic secretion [31]. Due to the diverse composition of fatty acid residues binding to the glycerol backbone, individual PCs and lyso-PCs have different functional properties and reactions to oxidative stress [32–35]. A reduction in the levels of several individual PC and lyso-PC species has previously been reported in patients with atherosclerosis in comparison to those of healthy subjects via a targeted metabolomics approach [36], indicating that abnormal glycerophospholipid metabolism might be correlated with the aberrant activity of several enzymes, such as PLA1, PLA2 and LCAT, crucial components of oxidized LDL (Ox-LDL), which contributes to atherogenesis [37, 38].

In our study, the levels of 13 glycerophospholipids (1 lyso-PC and 12 PCs) and 9 glycerophospholipids (2 lyso-PC and 7 PCs) were observed to be significantly lower in the upper serum Hp tertile group than in the lower tertile group in T2DM patients and non-DM subjects, respectively. As reported previously, it was speculated that the influence of Hp on the cholesterol level might be related to the ability of Hp to bind apolipoprotein E, which results in the displacement and inhibition of the LCAT cholesterol esterification rate [18]. The decrease in levels of PC and lyso-PC species in patients with higher serum Hp concentrations, which might be caused by aberrant enzyme activity, provide a likely explanation for the potential mechanism underlying the interaction of serum Hp and lipid metabolism.

One highlight of the glycerophospholipids associated with serum Hp is PC ae C32:2, which was shown to be significantly correlated with carotid IAD in T2DM patients. Carotid IAD and IMT measured by ultrasound serve as a non-invasive surrogate for assessing the atherosclerotic process [39, 40]. Although carotid IMT is recommended for early detection and cardiovascular risk evaluation, the role of carotid IAD in cardiovascular outcomes prediction has gradually been recognized [41, 42]. Several studies observed that common carotid IAD and not carotid IMT was correlated with the prevalence of cardiovascular events and might have predictive value for cardiovascular outcomes [43, 44]. In our research, metabolite PC ae C32:2 was correlated with serum Hp and common carotid IAD in T2DM patients with a median diabetes duration of 10 years. We speculated that the lack of associations between metabolites and carotid IMT might due to several factors. One factor to take into consideration is vascular remodelling. With the deposition and development of atherosclerotic plaques, vascular remodelling occurs and results in increased carotid diameter, called the Glagov phenomenon [45]. As a sum of the lumen diameter and of IMT, carotid IAD is associated with carotid IMT, left ventricular mass and cardiovascular risk factors, which is more obvious to reflect the change of carotid arterial functions [46]. The other is the possibility that carotid IMT is more sensitive to metabolites in those patients with mild impaired glucose regulation or lower burdens of subclinical cardiovascular diseases than those with larger burden T2DM patients. Further studies should be carried out to cast insights into the mechanism behind the association of serum Hp and carotid arterial functions.

Interestingly, changes in the ratios between two single metabolites might reflect biological situations such as alterations in enzyme activities or imbalances in metabolic pathways relevant for a certain phenotype [47–49]. A study reported recently that the metabolite ratio of Val/PC ae C32:2 was not only associated with measures of oral glucose tolerance test -derived β cell function and insulin resistance but also correlated with an increased risk of T2DM [27]. The observed effects, independent of currently known risk factors, were stronger than those of the individual metabolites, indicating that the application of ratios might improve prediction above that of single metabolites.

Based on this background, the metabolite ratios of 21 amino acids to PC ae C32:2 were compared in this study to evaluate the association with serum Hp levels and clinical traits. As a result, we identified that the metabolite ratios of two acidic amino acids, including Asp/PC ae C32:2 and Glu/PC ae C32:2 were significantly correlated with serum Hp levels, carotid IMT or IAD, and other lipid metabolic parameters after adjusting for confounding factors. As reported previously, acidic amino acids, including aspartate and glutamate were identified to have an intrinsic peroxidase-like activity, which plays a key role in lipid oxidation [50]. It was speculated that metabolites found in our study might mediate the aberrant activity of several enzymes caused by the action of Hp binding to apolipoprotein E and apolipoprotein A-I, which disturbs cholesterol esterification and lipid oxidation, contributing to blood vessel endothelial dysfunction and atherogenesis [37, 38]. Changes in those metabolite ratios might reflect the alterations in enzyme activities relevant to the rate-limiting step in glycolipid catabolism [51, 52]. In view of this, we speculated that it might be useful for early identification of subjects with an increased risk of cardio-cerebrovascular diseases or helpful to identify potential drug targets for new therapies if verified by further prospective studies [53–55].

To the best of our knowledge, this is the first study to use a targeted metabolomics approach to analyse blood metabolites in relation to serum Hp levels and carotid arterial functions. However, there are several limitations in this research. First, the sample we recruited to perform metabolite measurements was relatively small, and large-scale studies are needed to confirm our results. Second, part of the explanation for our results is limited by the targeted metabolomics kit we used, as it does not provide a detailed analysis of the lipid composition of metabolites, such as PC ae C32:2. Its exact role in the physiology and molecular aetiology awaits examination in further functional studies. Third, as carotid IMT is recommended for early detection and cardiovascular risk evaluation in general population, carotid IAD was not recorded in non-DM subjects in this study. The association between serum Hp levels and carotid arterial functions should be evaluated in other complex diseases. Finally, a direct causal relationship cannot be established in view of the cross-sectional design of this study. Therefore, the findings identified in our study should be further validated in a prospective study.

## Conclusions

To summarize, we show that a number of blood metabolites, especially PCs and lyso-PCs, were associated with serum Hp levels and blood lipid metabolism by using a targeted metabolomics approach in Chinese T2DM patients and non-DM subjects. This finding provides a new insight into potential mechanisms behind the interaction between serum Hp and carotid arterial functions. Furthermore, the metabolite ratios of two acidic amino acids, including Asp/PC ae C32:2 and Glu/PC ae C32:2 may be used to predict early risk of cardio-cerebrovascular diseases if validated in further studies.

## Additional file

**Additional file 1: Table S1.** Correlation between clinical traits and serum Hp levels in T2DM patients. **Table S2.** Correlation between clinical traits and serum Hp levels in non-DM subjects. **Table S3.** Blood metabolites associated with serum Hp levels. **Table S4.** Metabolite ratios correlated with clinical traits in T2DM patients. **Table S5.** Metabolite ratios correlated with clinical traits in non-DM subjects. **Figure S1.** Serum Hp levels in non-diabetes mellitus subjects.

## Abbreviations

T2DM: type 2 diabetes mellitus
Non-DM: non-diabetes mellitus
Hp: haptoglobin
IMT: intima-media thickness
IAD: inter-adventitial diameter
BMI: body mass index
HbA1c: haemoglobin A1c
HDL-C: high-density lipoprotein-cholesterol
LDL-C: low-density lipoprotein-cholesterol
lyso-PCs: lyso-phosphatidylcholines
PCs: phosphatidylcholines
UPLC: ultra performance liquid chromatography
μM: μmol/L
ELISA: enzyme-linked immunosorbent assay
ANOVA: analysis of variance
Hb–Hp: haemoglobin–haptoglobin
PLA1: phospholipase A1
PLA2: phospholipase A2
LCAT: lecithin–cholesterol acyltransferase
Ox-LDL: oxidized LDL
