# Supplementary material for: Association between serum haptoglobin and carotid arterial functions: usefulness of a targeted metabolomics approach
Source: Cardiovasc Diabetol. 2019 Jan 11;18:8. doi: 10.1186/s12933-019-0808-2 (PMC6329046; doi:10.1186/s12933-019-0808-2)
Supplement: Supplementary file 1 — Additional file 1: Table S1. Correlation between clinical traits and serum Hp levels in T2DM patients. Table S2. Correlation between clinical traits and serum Hp levels in non-DM subjects. Table S3. Blood metabolites associated with serum Hp levels. Table S4. Metabolite ratios correlated with clinical traits in T2DM patients. Table S5. Metabolite ratios correlated with clinical traits in non-DM subjects. Figure S1. Serum Hp levels in non-diabetes mellitus subjects. [file 12933_2019_808_MOESM1_ESM.docx]

**Supplemental** **Table S1**

**Correlation between clinical traits and serum Hp levels in T2DM patients**

| **Variable** | **β coefficient** | **SE** | ***P* value** |
| --- | --- | --- | --- |
| Total cholesterol | -0.1210 | 0.0982 | 0.2202 |
| Triglycerides | -0.0642 | 0.0992 | 0.5191 |
| High density lipoprotein-cholesterol | -0.0494 | 0.0301 | 0.1038 |
| Low density lipoprotein-cholesterol | -0.0574 | 0.0835 | 0.4938 |
| Carotid intima-media thickness | 0.0027 | 0.0175 | 0.8798 |
| Carotid inter-adventitial diameter | 0.2193 | 0.0750 | **0.0042** |

Hp: haptoglobin; T2DM: type 2 diabetes mellitus; Multiple linear regression was performed to evaluate the correlation between clinical traits and serum Hp levels tertiles with adjusting for age, sex, body mass index, blood pressure, duration of diabetes and HbA1c levels. *P* values < 0.05 are shown in bold.

**Supplemental Table S2**

**Correlation between clinical traits and serum Hp levels in non-DM subjects**

| **Variable** | **β coefficient** | **SE** | ***P* value** |
| --- | --- | --- | --- |
| Total cholesterol | 0.0032 | 0.0059 | 0.5843 |
| Triglycerides | -0.0134 | 0.0161 | 0.3942 |
| High density lipoprotein-cholesterol | 0.0006 | 0.0100 | 0.9503 |
| Low density lipoprotein-cholesterol | 0.0056 | 0.0092 | 0.5447 |
| Carotid intima-media thickness | 0.0038 | 0.0070 | 0.5898 |

Hp: haptoglobin; Non-DM: non-diabetes mellitus; Multiple linear regression was performed to evaluate the correlation between clinical traits and serum Hp levels tertiles with adjusting for age, sex, body mass index, blood pressure and HbA1c levels. *P* values < 0.05 are shown in bold.

**Supplemental Table S3**

**Blood metabolites associated with serum Hp levels**

| **Metabolites** | **β coefficient** | **SE** | ***P* Value** |
| --- | --- | --- | --- |
| **T2DM (n = 120)** |  |  |  |
| Acylcarnitines (*μM*) |  |  |  |
| C12:1 | -3.2823 | 1.1806 | 0.0063 |
| Lyso-phosphatidylcholines (*μM*) | |  |  |
| lysoPC a C18:1 | -0.0461 | 0.0160 | 0.0046 |
| lysoPC a C18:2 | -0.0159 | 0.0075 | 0.0364 |
| lysoPC a C20:3 | -0.3806 | 0.1314 | 0.0045 |
| Phosphatidylcholines (*μM*) |  |  |  |
| PC aa C32:0 | -0.0635 | 0.0291 | 0.0311 |
| PC aa C34:1 | -0.0035 | 0.0015 | 0.0227 |
| PC aa C36:0 | -0.1733 | 0.0685 | 0.0127 |
| PC aa C36:1 | -0.0189 | 0.0085 | 0.0278 |
| PC aa C36:5 | -0.0235 | 0.0112 | 0.0382 |
| PC aa C38:0 | -0.2334 | 0.0742 | 0.0021 |
| PC aa C38:5 | -0.0165 | 0.0065 | 0.0128 |
| PC aa C38:6 | -0.0082 | 0.0027 | 0.0030 |
| PC aa C40:2 | -1.3112 | 0.5175 | 0.0126 |
| PC aa C40:3 | -1.0445 | 0.5018 | 0.0396 |
| PC aa C42:2 | -1.5933 | 0.5658 | 0.0057 |
| PC aa C42:5 | -1.9466 | 0.6380 | 0.0028 |
| PC aa C42:6 | -1.3745 | 0.5514 | 0.0141 |
| PC ae C32:1 | -0.5327 | 0.1775 | 0.0033 |
| PC ae C32:2 | -2.9892 | 0.6370 | <0.0001 |
| PC ae C34:0 | -0.8997 | 0.3549 | 0.0125 |
| PC ae C34:1 | -0.1897 | 0.0714 | 0.0090 |
| PC ae C34:2 | -0.0821 | 0.0350 | 0.0207 |
| PC ae C34:3 | -0.1396 | 0.0462 | 0.0031 |
| PC ae C36:0 | -0.8638 | 0.3399 | 0.0123 |
| PC ae C36:1 | -0.1277 | 0.0541 | 0.0198 |
| PC ae C36:3 | -0.1461 | 0.0580 | 0.0131 |
| PC ae C36:5 | -0.0668 | 0.0276 | 0.0170 |
| PC ae C38:0 | -0.4619 | 0.1719 | 0.0082 |
| PC ae C38:5 | -0.0488 | 0.0243 | 0.0472 |
| PC ae C38:6 | -0.1298 | 0.0406 | 0.0018 |
| PC ae C40:1 | -0.4949 | 0.2018 | 0.0156 |
| PC ae C40:2 | -0.4747 | 0.2320 | 0.0430 |
| PC ae C40:5 | -0.2821 | 0.1086 | 0.0106 |
| PC ae C40:6 | -0.1960 | 0.0766 | 0.0118 |
| PC ae C42:0 | -1.4768 | 0.4611 | 0.0017 |
| PC ae C42:3 | -0.9873 | 0.4333 | 0.0245 |
| PC ae C44:4 | -2.1018 | 0.8997 | 0.0212 |
| Amino Acids (*μM*) |  |  |  |
| His | -0.0066 | 0.0032 | 0.0423 |
| Trp | -0.0080 | 0.0038 | 0.0391 |
| Tyr | -0.0055 | 0.0028 | 0.0485 |
| Ac-Orn | 4.4704 | 2.2407 | 0.0487 |
| **Non-DM (n = 120)** |  |  |  |
| C12:1 | 1.5834 | 0.6137 | 0.0111 |
| C16 | 1.4847 | 0.6070 | 0.0159 |
| C4:1 | 18.7529 | 7.2090 | 0.0105 |
| Lyso-phosphatidylcholines (*μM*) | |  |  |
| lysoPC a C17:0 | -0.2295 | 0.1070 | 0.0340 |
| Phosphatidylcholines (*μM*) |  |  |  |
| PC aa C32:3 | -1.8031 | 0.6213 | 0.0044 |
| PC ae C32:1 | -0.2696 | 0.1068 | 0.0129 |
| PC ae C32:2 | -0.9771 | 0.3309 | 0.0038 |
| PC ae C34:3 | -0.0634 | 0.0316 | 0.0468 |
| PC ae C36:2 | -0.0545 | 0.0225 | 0.0169 |
| PC ae C38:0 | -0.3027 | 0.1136 | 0.0088 |
| PC ae C38:3 | -0.1838 | 0.0741 | 0.0145 |
| PC ae C38:6 | -0.0674 | 0.0306 | 0.0297 |
| PC ae C40:1 | -0.4872 | 0.1452 | 0.0011 |
| PC ae C42:1 | -1.3505 | 0.4068 | 0.0012 |
| Amino Acids (*μM*) |  |  |  |
| Gly | -0.0006 | 0.0003 | 0.0237 |
| Orn | -0.0010 | 0.0005 | 0.0304 |
| Trp | -0.0075 | 0.0036 | 0.0390 |

Hp: haptoglobin; T2DM: type 2 diabetes mellitus; Non-DM: non-diabetes mellitus; His: histidine; Trp: tryptophan; Tyr: tyrosine; Ac-Orn: acyl-Ornithine; Gly: Glycine; Orn: Ornithine; Linear regression was performed to evaluate the correlation between blood metabolites and serum Hp levels. Blood metabolites as the dependent variable and Log (serum Hp levels) as independent variables. *P* values < 0.05 are shown in table.

**Supplemental Table S4**

**Metabolite ratios correlated with clinical traits in T2DM patients**

| **Metabolite ratio** | **Total cholesterol** | **Triglycerides** | **HDL-C** | **LDL-C** | **Serum Hp** | **Carotid IMT** | **Carotid IAD** |
| --- | --- | --- | --- | --- | --- | --- | --- |
| Ala/PC ae C32:2 | -1.145 ± 0.414 | 0.274 ± 0.094 | 0.158 ± 0.195 | 0.352 ± 0.234 | 0.037 ± 0.017 | 0.182 ± 0.163 | -0.260 ± 0.279 |
|  | **0.0066** | **0.0043** | 0.4202 | 0.1350 | **0.0288** | 0.2654 | 0.3529 |
| Arg/PC ae C32:2 | -0.243 ± 0.396 | 0.001 ± 0.090 | -0.100 ± 0.187 | -0.133 ± 0.223 | 0.035 ± 0.016 | 0.284 ± 0.155 | -0.345 ± 0.267 |
|  | 0.5408 | 0.9908 | 0.5935 | 0.5529 | **0.0276** | 0.0710 | 0.1994 |
| Cit/PC ae C32:2 | 0.133 ± 0.477 | -0.120 ± 0.108 | -0.254 ± 0.225 | -0.353 ± 0.270 | 0.034 ± 0.019 | 0.274 ± 0.188 | -0.427 ± 0.322 |
|  | 0.7818 | 0.2708 | 0.2613 | 0.1935 | 0.0775 | 0.1464 | 0.1876 |
| Gln/PC ae C32:2 | -1.052 ± 0.320 | 0.082 ± 0.073 | 0.048 ± 0.151 | 0.230 ± 0.181 | 0.027 ± 0.013 | 0.180 ± 0.126 | -0.291 ± 0.216 |
|  | **0.0014** | 0.2590 | 0.7515 | 0.2053 | **0.0350** | 0.1549 | 0.1809 |
| Gly/PC ae C32:2 | -0.825 ± 0.353 | 0.041 ± 0.080 | -0.006 ± 0.167 | 0.136 ± 0.200 | 0.026 ± 0.014 | 0.161 ± 0.139 | -0.282 ± 0.238 |
|  | **0.0214** | 0.6133 | 0.9721 | 0.4973 | 0.0689 | 0.2492 | 0.2396 |
| His/PC ae C32:2 | -1.281 ± 0.283 | 0.216 ± 0.064 | 0.092 ± 0.134 | 0.496 ± 0.160 | 0.022 ± 0.011 | 0.168 ± 0.111 | -0.506 ± 0.191 |
|  | **<.0001** | **0.0011** | 0.4948 | **0.0025** | 0.0566 | 0.1352 | **0.0093** |
| Leu/PC ae C32:2 | -0.999 ± 0.300 | 0.205 ± 0.068 | -0.002 ± 0.142 | 0.462 ± 0.169 | 0.035 ± 0.012 | 0.216 ± 0.118 | -0.386 ± 0.202 |
|  | **0.0012** | **0.0033** | 0.9910 | **0.0075** | **0.0044** | 0.0692 | 0.0591 |
| Lys/PC ae C32:2 | -0.889 ± 0.298 | 0.135 ± 0.068 | -0.058 ± 0.141 | 0.348 ± 0.168 | 0.022 ± 0.012 | 0.264 ± 0.117 | -0.706 ± 0.201 |
|  | **0.0035** | **0.0484** | 0.6809 | **0.0411** | 0.0655 | **0.0263** | **0.0007** |
| Met/PC ae C32:2 | -0.749 ± 0.321 | 0.061 ± 0.073 | -0.033 ± 0.151 | 0.196 ± 0.181 | 0.015 ± 0.013 | 0.286 ± 0.126 | -0.256 ± 0.217 |
|  | **0.0214** | 0.4063 | 0.8302 | 0.2829 | 0.2354 | **0.0252** | 0.2394 |
| Orn/PC ae C32:2 | -0.510 ± 0.486 | 0.017 ± 0.110 | -0.232 ± 0.230 | 0.084 ± 0.275 | 0.010 ± 0.020 | 0.041 ± 0.191 | -0.562 ± 0.328 |
|  | 0.2963 | 0.8795 | 0.3147 | 0.7597 | 0.6236 | 0.8293 | 0.0897 |
| Phe/PC ae C32:2 | -0.849 ± 0.297 | 0.115 ± 0.067 | -0.092 ± 0.140 | 0.379 ± 0.168 | 0.021 ± 0.012 | 0.338 ± 0.117 | -0.276 ± 0.201 |
|  | **0.0052** | 0.0918 | 0.5156 | **0.0260** | 0.0867 | **0.0047** | 0.1722 |
| Pro/PC ae C32:2 | -0.811 ± 0.437 | 0.173 ± 0.099 | -0.136 ± 0.206 | 0.144 ± 0.247 | 0.024 ± 0.018 | 0.012 ± 0.172 | -0.269 ± 0.295 |
|  | 0.0661 | 0.0841 | 0.5106 | 0.5607 | 0.1711 | 0.9465 | 0.3633 |
| Ser/PC ae C32:2 | -0.786 ± 0.323 | -0.003 ± 0.073 | -0.210 ± 0.153 | 0.288 ± 0.183 | 0.023 ± 0.013 | 0.106 ± 0.127 | -0.446 ± 0.218 |
|  | **0.0166** | 0.9676 | 0.1715 | 0.1178 | 0.0808 | 0.4057 | **0.0434** |
| Thr/PC ae C32:2 | -1.263 ± 0.337 | 0.135 ± 0.076 | -0.010 ± 0.159 | 0.364 ± 0.190 | 0.031 ± 0.014 | 0.075 ± 0.132 | -0.309 ± 0.227 |
|  | **0.0003** | 0.0791 | 0.9506 | 0.0581 | **0.0240** | 0.5738 | 0.1769 |
| Trp/PC ae C32:2 | -0.941 ± 0.325 | 0.199 ± 0.074 | 0.070 ± 0.153 | 0.383 ± 0.183 | 0.018 ± 0.013 | 0.288 ± 0.128 | -0.440 ± 0.219 |
|  | **0.0046** | **0.0079** | 0.6494 | **0.0391** | 0.1668 | **0.0263** | **0.0474** |
| Tyr/PC ae C32:2 | -0.844 ± 0.316 | 0.112 ± 0.072 | -0.075 ± 0.149 | 0.371 ± 0.178 | 0.013 ± 0.013 | 0.222 ± 0.124 | -0.410 ± 0.213 |
|  | 0.0087 | 0.1206 | 0.6142 | **0.0398** | 0.2936 | 0.0763 | 0.0573 |

T2DM: type 2 diabetes mellitus; Ala: alanine; Arg: arginine; Cit: citrulline; Gln: glutanine; Gly: glicine; His: histidine; Leu: leucine; Lys: lysine; Met: methionine; Orn: ornithine; Phe: phenylalanine; Pro: proline; Ser: serine; Thr: threonine; Trp: tryptophan; Tyr: tyrosine; Hp: haptoglobin; HDL-C: high density lipoprotein-cholesterol; LDL-C: low density lipoprotein-cholesterol; IMT: intima-media thickness; IAD: inter-adventitial diameter. Multiple linear regression analysis was applied to perform the analysis after adjusting for age, sex and body mass index with total cholesterol, triglycerides, HDL-C, LDL-C, serum Hp levels tertiles, carotid IMT and IAD as independent variables. β ± SE and *P* values are shown in table. *P* values < 0.05 are shown in bold.

**Supplemental Table S5**

**Metabolite ratios correlated with clinical traits in Non-DM subjects**

| **Metabolite ratio** | **Total cholesterol** | **Triglycerides** | **HDL-C** | **LDL-C** | **Serum Hp** | **Carotid IMT** |
| --- | --- | --- | --- | --- | --- | --- |
| Ala/PC ae C32:2 | -0.344 ± 0.524 | 0.173 ± 0.098 | -0.302 ± 0.215 | 0.121 ± 0.307 | 0.025 ± 0.014 | 0.208 ± 0.200 |
|  | 0.5123 | 0.0792 | 0.1625 | 0.6929 | 0.0763 | 0.3023 |
| Arg/PC ae C32:2 | 0.197 ± 0.463 | -0.010 ± 0.086 | -0.502 ± 0.190 | -0.134 ± 0.271 | 0.036 ± 0.012 | 0.139 ± 0.177 |
|  | 0.6717 | 0.9110 | **0.0094** | 0.6220 | **0.0048** | 0.4357 |
| Asn/PC ae C32:2 | 1.149 ± 0.540 | -0.136 ± 0.101 | -0.668 ± 0.221 | -0.817 ± 0.316 | 0.008 ± 0.015 | 0.116 ± 0.207 |
|  | 0.0357 | 0.1801 | **0.0032** | **0.0111** | 0.5727 | 0.5746 |
| Cit/PC ae C32:2 | 0.524 ± 0.652 | -0.125 ± 0.122 | -0.496 ± 0.267 | -0.209 ± 0.381 | 0.001 ± 0.018 | 0.143 ± 0.249 |
|  | 0.4228 | 0.3084 | 0.0661 | 0.5844 | 0.9879 | 0.5670 |
| Gln/PC ae C32:2 | 0.455 ± 0.493 | -0.092 ± 0.092 | -0.595 ± 0.202 | -0.320 ± 0.289 | 0.005 ± 0.013 | 0.141 ± 0.189 |
|  | 0.3587 | 0.3217 | **0.0040** | 0.2706 | 0.7223 | 0.4580 |
| Gly/PC ae C32:2 | -0.093 ± 0.661 | -0.076 ± 0.123 | -0.338 ± 0.271 | -0.011 ± 0.387 | 0.008 ± 0.018 | 0.352 ± 0.253 |
|  | 0.8882 | 0.5417 | 0.2142 | 0.9768 | 0.6464 | 0.1674 |
| His/PC ae C32:2 | 0.083 ± 0.456 | -0.054 ± 0.085 | -0.491 ± 0.187 | -0.129 ± 0.267 | 0.015 ± 0.012 | 0.301 ± 0.175 |
|  | 0.8556 | 0.5248 | **0.0099** | 0.6292 | 0.2341 | 0.0875 |
| Ile/PC ae C32:2 | 0.335 ± 0.537 | 0.089 ± 0.100 | -0.758 ± 0.220 | -0.161 ± 0.315 | 0.003 ± 0.014 | 0.382 ± 0.206 |
|  | 0.5346 | 0.3783 | **0.0008** | 0.6103 | 0.8318 | 0.0661 |
| Leu/PC ae C32:2 | 1.797 ± 0.758 | 0.022 ± 0.142 | -1.058 ± 0.310 | -0.965 ± 0.444 | 0.018 ± 0.020 | 0.556 ± 0.290 |
|  | **0.0196** | 0.8765 | **0.0009** | **0.0318** | 0.3780 | 0.0577 |
| Lys/PC ae C32:2 | 0.675 ± 0.539 | -0.081 ± 0.101 | -0.615 ± 0.221 | -0.270 ± 0.315 | 0.012 ± 0.015 | 0.301 ± 0.206 |
|  | 0.2134 | 0.4229 | **0.0063** | 0.3938 | 0.4251 | 0.1469 |
| Met/PC ae C32:2 | 0.417 ± 0.492 | -0.084 ± 0.092 | -0.559 ± 0.202 | -0.319 ± 0.288 | 0.021 ± 0.013 | 0.315 ± 0.188 |
|  | 0.3987 | 0.3607 | **0.0066** | 0.2709 | 0.1095 | 0.0979 |
| Orn/PC ae C32:2 | 0.165 ± 0.727 | 0.015 ± 0.136 | -0.712 ± 0.298 | -0.412 ± 0.426 | -0.011 ± 0.020 | 0.156 ± 0.278 |
|  | 0.8206 | 0.9147 | **0.0187** | 0.3358 | 0.5892 | 0.5768 |
| Pro/PC ae C32:2 | 0.687 ± 0.728 | 0.212 ± 0.136 | -0.832 ± 0.298 | -0.657 ± 0.426 | 0.026 ± 0.020 | 0.133 ± 0.279 |
|  | 0.3476 | 0.1219 | **0.0063** | 0.1261 | 0.1895 | 0.6341 |
| Ser/PC ae C32:2 | 0.213 ± 0.521 | -0.157 ± 0.097 | -0.720 ± 0.213 | -0.306 ± 0.305 | 0.019 ± 0.014 | 0.097 ± 0.199 |
|  | 0.6841 | 0.1107 | **0.0010** | 0.3187 | 0.1840 | 0.6275 |
| Thr/PC ae C32:2 | 0.471 ± 0.577 | -0.081 ± 0.108 | -0.726 ± 0.237 | -0.467 ± 0.338 | 0.009 ± 0.016 | 0.118 ± 0.221 |
|  | 0.4167 | 0.4554 | **0.0028** | 0.1702 | 0.5681 | 0.5941 |
| Trp/PC ae C32:2 | 0.187 ± 0.436 | 0.031 ± 0.082 | -0.567 ± 0.179 | -0.121 ± 0.255 | 0.003 ± 0.012 | 0.258 ± 0.167 |
|  | 0.6691 | 0.7050 | 0.0020 | 0.6357 | 0.7734 | 0.1247 |
| Tyr/PC ae C32:2 | 0.596 ± 0.479 | -0.111 ± 0.089 | -0.751 ± 0.196 | -0.436 ± 0.280 | 0.018 ± 0.013 | 0.259 ± 0.183 |
|  | 0.2158 | 0.2189 | **0.0002** | 0.1227 | 0.1608 | 0.1602 |
| Val/PC ae C32:2 | 0.190 ± 0.479 | 0.049 ± 0.09 | -0.686 ± 0.196 | -0.089 ± 0.281 | 0.016 ± 0.013 | 0.237 ± 0.183 |
|  | 0.6922 | 0.5867 | 0.0007 | 0.7517 | 0.2063 | 0.1984 |

Non-DM: non-diabetes mellitus; Ala: alanine; Arg: arginine; Asn: asparagine; Cit: citrulline; Gln: glutanine; Gly: glicine; His: histidine; Ile: isoleucine; Leu: leucine; Lys: lysine; Met: methionine; Orn: ornithine; Pro: proline; Ser: serine; Thr: threonine; Trp: tryptophan; Tyr: tyrosine; Val: valine; Hp: haptoglobin; HDL-C: high density lipoprotein-cholesterol; LDL-C: low density lipoprotein-cholesterol; IMT: intima-media thickness. Multiple linear regression analysis was applied to perform the analysis after adjusting for age, sex and body mass index with total cholesterol, triglycerides, HDL-C, LDL-C, serum Hp levels tertiles and carotid IMT as independent variables. β ± SE and *P* values are shown in table. *P* values < 0.05 are shown in bold.

**Supplemental Figure S1**


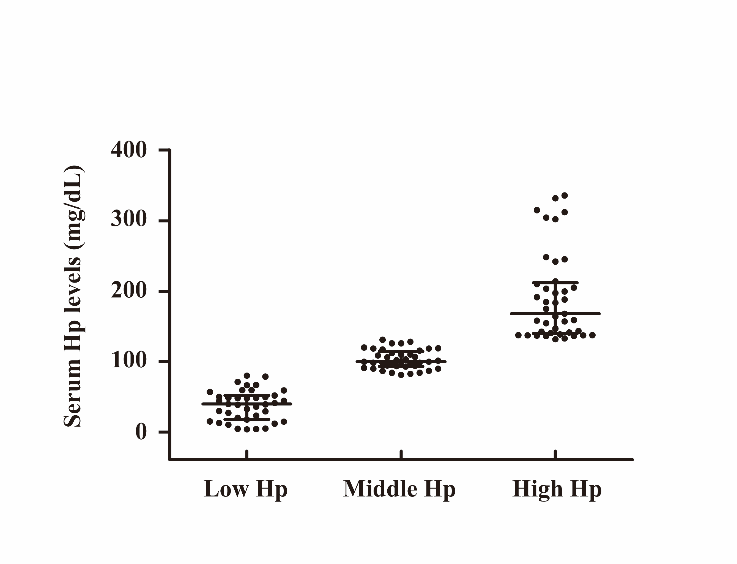


Fig. S1 Serum Hp levels in non-diabetes mellitus subjects. Hp: haptoglobin; It shows a comparison of serum Hp levels among Hp tertiles, *P* < 0.0001; the serum Hp levels are shown in dot plots; the median is indicated by the middle black solid line. The lower and upper quartiles are shown by the bottom and top black solid lines, respectively.
